# Differentiation of insulinoma from accessory spleen by ^99m^Tc-labelled heat-denaturated red blood cell scintigraphy: case report

**DOI:** 10.1186/s12902-020-00671-9

**Published:** 2021-01-07

**Authors:** Emre Gezer, Berrin Çetinarslan, Dilek Karakaya, Zeynep Cantürk, Alev Selek, Mehmet Sözen, Serkan İşgören

**Affiliations:** 1 Faculty of Medicine, Department of Endocrinology and Metabolism, Kocaeli University, Kocaeli, Turkey; 2 Gebze MedicalPark Hospital, Division of Endocrinology and Metabolism, Kocaeli, Turkey; 3 Faculty of Medicine, Department of Nuclear Medicine, Kocaeli University, Kocaeli, Turkey

**Keywords:** Insulinoma, Accessory spleen, ^68^Ga-DOTATATE PET/CT, ^99m^Tc-HDRBC scintigraphy-SPECT/CT, Case report

## Abstract

**Background:**

Neuroendocrine tumors (NETs) are rare tumors harboring overexpression of somatostatin receptors (SSTRs) on their cell membrane. Because some organs, such as the spleen, adrenal glands and liver, physiologically express SSTR, it might be challenging to distinguish some pancreatic NETs located in the pancreatic tail from the accessory spleen next to the splenic hilum. In this manuscript, we report a case with hypoglycemia attack and 2 different masses displayed by Gallium 68-tetraazacyclododecane tetraacetic acid-octreotate (^68^Ga-DOTATATE) positron emission tomography/computed tomography (PET/CT).

**Case presentation:**

A 63-year-old woman presented to the hospital with confusion and profuse sweating. Biochemical diagnosis of insulinoma was established. ^68^Ga-DOTATATE PET/CT revealed two masses with increased tracer uptake located adjacent to the splenic hilum and inferior pole of the spleen which were initially reported as two separate accessory spleens. Then, ^99m^Tc-labelled heat-denaturated red blood cell (^99m^Tc-HDRBC) scintigraphy-single-photon emission computed tomography (SPECT)/CT was performed to distinguish a NET in the pancreatic tail from accessory spleen at the splenic hilum. Enhanced tracer uptake remained in the inferior pole of spleen, but not in the splenic hilum. The lesions were suggestive of insulinoma in the pancreatic tail and an accessory spleen adjacent to the inferior pole of the spleen.

**Conclusion:**

Approximately 10% of the population have an accessory spleen which can show similar imaging characteristics with pancreatic NETs, especially if located in the pancreatic tail. In our presented case, ^99m^Tc-HDRBC scintigraphy-SPECT/CT is a useful nuclear medicine method to differentiate a NET in the pancreatic tail from accessory spleen at the splenic hilum which may avoid unnecessary surgeries in the presence of enhanced tracer uptake or vice versa.

## Key messages

^99m^Tc-HDRBC scintigraphy-SPECT/CT is a useful nuclear medicine method to differentiate a NET in the pancreatic tail from accessory spleen at the splenic hilum which may avoid unnecessary surgeries in the presence of enhanced tracer uptake or vice versa.

## Background

Neuroendocrine tumors (NETs) are rare tumors harboring overexpression of somatostatin receptors (SSTRs) on their cell membrane and insulinoma is the most common functioning NET, with an incidence of 1–4 cases per a million person years [1, 2]. Insulinomas are generally benign lesions which can be curative in most patients following surgical resection [3], indicating the preoperative localization of the lesions as one of the mandatory steps in the management of patients with insulinoma. Similar to other ^68^Gallium (Ga)-labeled analogs of octreotide, such as tetraazacyclododecane-tetraacetic acid (DOTA)-d-Phe1-Tyr3-octreotide (DOTATOC) and DOTA-1-Nal3-octreotide (DOTANOC), DOTA-d-Phe1-Tyr3-octreotate (DOTATATE) is a radiopharmaceutical which binds to SSTR type 2, while having less affinity for SSTR type 3 and 5, compared to the other two radiopharmaceuticals [4].

^68^Ga-DOTA-d-Phe1-Tyr3-octreotate (^68^Ga-DOTATATE) positron emission tomography/computed tomography (PET/CT) is an important imaging modality in diagnosis, localizing and staging of NETs [5]. It might be challenging to distinguish some pancreatic NETs located in the pancreatic tail from the accessory spleen next to the splenic hilum, since some organs, such as the spleen, adrenal glands and liver, physiologically express SSTR [6]. In this manuscript, we report a case with hypoglycemia attack and 2 different masses with close proximity to the spleen which were initially reported as accessory spleen by ^68^Ga-DOTATATE PET/CT.

## Case presentation

A 63-year-old Caucasian woman with a 15-year history of hypertension presented to the emergency department with confusion and profuse sweating. She noted that her symptoms had first appeared mildly 5 years prior to admission. Both her parents had a history of hypertension and no history of any endocrinopathies. Physical examination revealed unremarkable findings, except a clouded consciousness. Her capillary blood glucose level was 45 mg/dL and a dextrose 5% infusion was started immediately. Within a short period of time, her consciousness was restored and capillary glucose level increased up to 110 mg/dL.

**Fig. 1:**
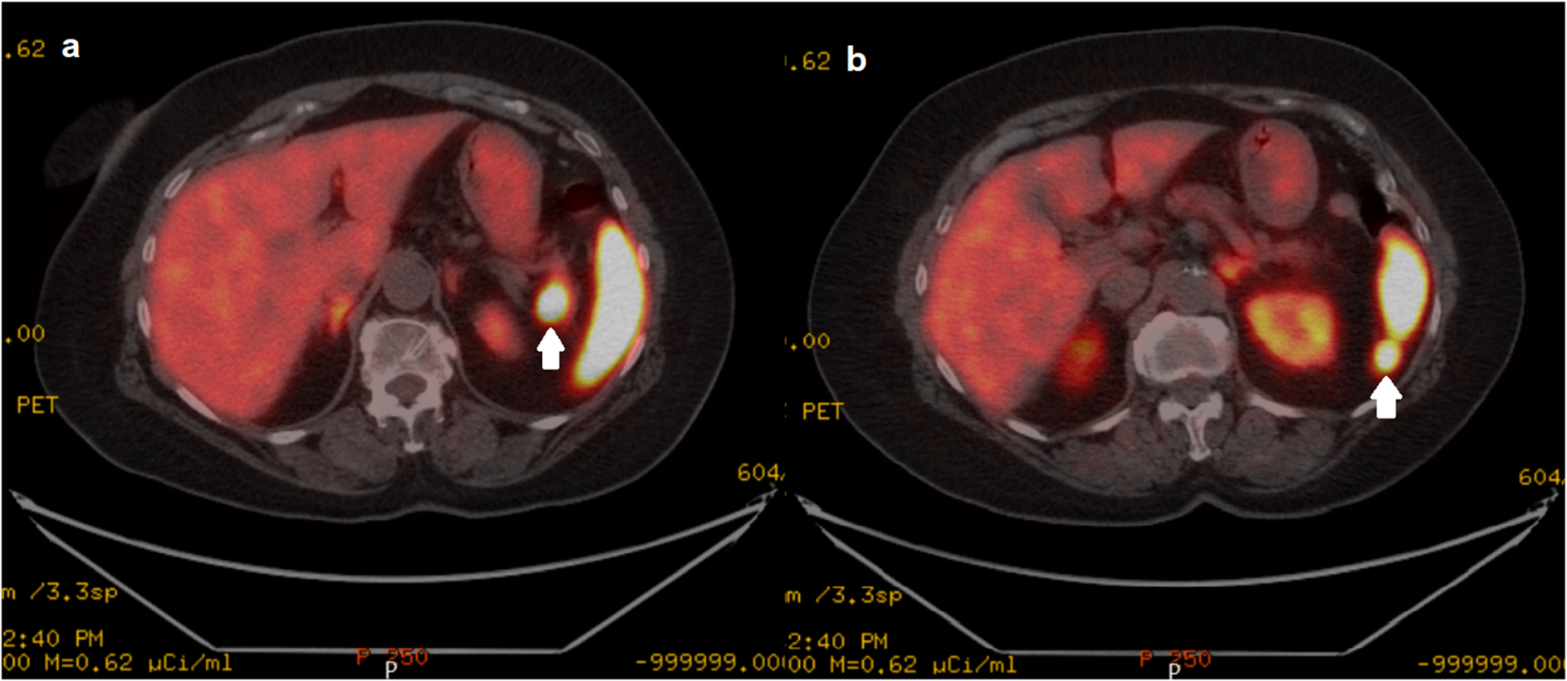
68Ga-DOTATATE PET/CT images, revealing two masses with intense tracer uptake in the pancreatic tail/splenic hilum (**a**) and in the inferior pole of the spleen (**b**)

**Fig. 2:**
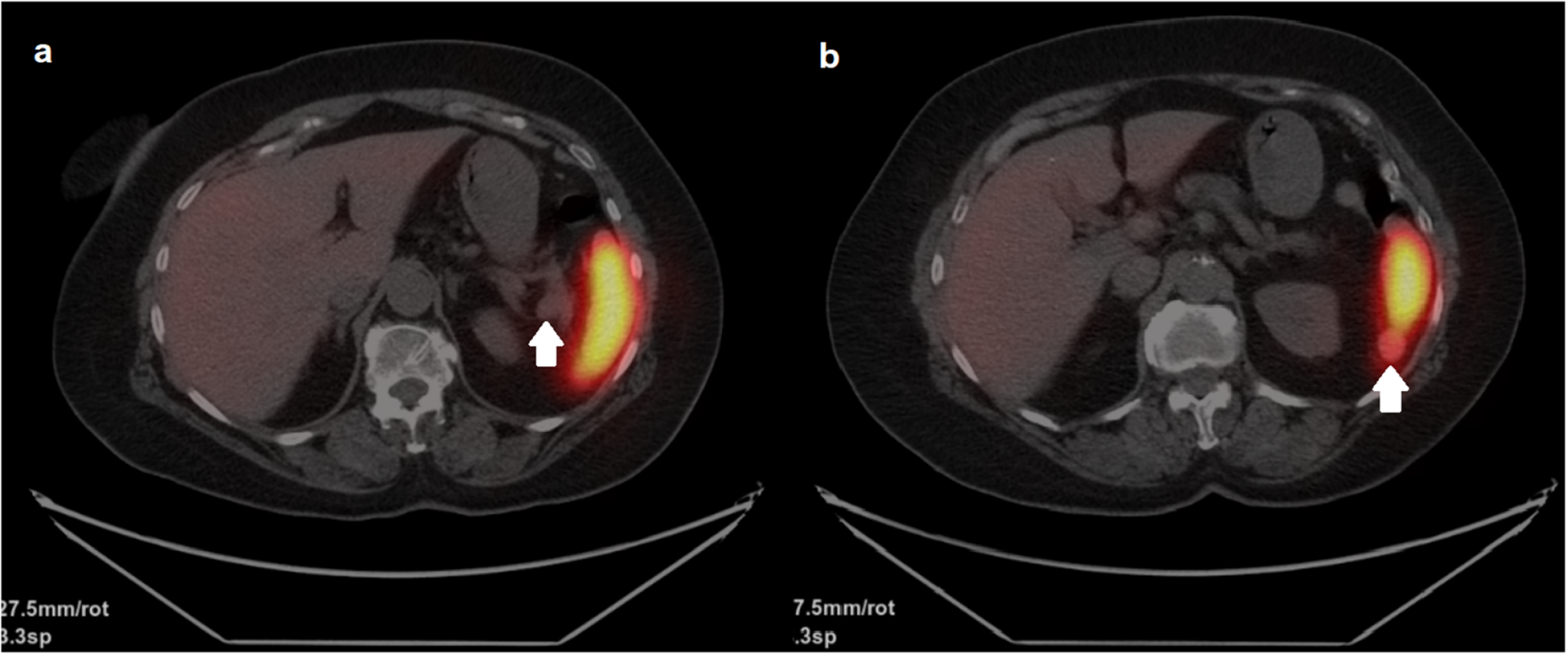
99mTc-HDRBC scintigraphy images, showing no tracer uptake in the pancreatic tail/splenic hilum (**a**), but remaining tracer uptake in the inferior pole of the spleen (**b**)

In the first hour after cessation of dextrose infusion, symptomatic hypoglycemia (plasma glucose: 31 mg/dL) developed and simultaneous insulin and C-peptide levels were found to be elevated, 41 μIU/ml (1.9–23 μIU/ml) and 12.7 ng/mL (0.9–7.1 ng/mL), respectively. The levels of antibodies to insulin were normal. Biochemical diagnosis of insulinoma was established and consequently, an abdominal CT was performed. No pathological findings were observed and an endoscopic ultrasonography (EUS) was planned, however it could not be performed due to some technical issues. As a result, ^68^Ga-DOTATATE PET/CT was performed. It revealed two masses with increased tracer uptake located adjacent to the splenic hilum and inferior pole of the spleen with the diameter of 1.6 cm and 1.8 cm, respectively. The lesions were initially reported as two separate accessory spleens due to their close proximity to the spleen and significantly elevated maximum standardized uptake value (SUVmax) within the lesions (SUVmax: 75.3 and 40.3, respectively) (Fig. 1). Then, ^99m^Tc-labelled heat-denaturated red blood cell (^99m^Tc-HDRBC) scintigraphy-single-photon emission computed tomography (SPECT)/CT was performed to distinguish a NET in the pancreatic tail from accessory spleen at the splenic hilum. Enhanced tracer uptake remained in the soft tissue lesion located in the inferior pole of the spleen, but not in the splenic hilum (Fig. 2). The lesions were suggestive of insulinoma in the pancreatic tail and an accessory spleen adjacent to the inferior pole of the spleen.

A hemipancreatectomy was planned; however, due to some intraoperative complications, splenectomy was also performed in addition to hemipancreatectomy. The histopathological findings revealed a grade 1 pancreatic neuroendocrine tumor with a maximum diameter of 16 mm and a Ki-67 proliferative index of 1% together with unremarkable splenic and accessory splenic tissue. On the first postoperative day, dextrose infusion was stopped and her plasma glucose level maintained within normal range during the hospital stay.

## Discussion

Accessory spleens arise from the left side of the dorsal mesogastrium during the embryological period and set apart from the main body of the spleen, most commonly located next to splenic hilum [7]. Approximately 10% of the population have an accessory spleen and the prevalence may increase up to 40% in patients undergoing autopsies [7, 8]. Accessory spleens can show similar imaging characteristics with pancreatic NETs, especially if located in the pancreatic tail [9]. In our case, biochemical diagnosis of insulinoma has been established; however, ^68^Ga-DOTATATE PET/CT demonstrated two masses located adjacent to the splenic hilum and inferior pole of the spleen, both considered as accessory spleens with similar tracer uptake.

^99m^Tc-HDRBC scintigraphy has been a beneficial imaging technique to detect gastrointestinal bleeding and to display the functional splenic tissue, [9] i.e., the differentiation of accessory spleen from NETs of pancreatic tail, estimating the function of auto-transplanted splenic tissue and determining the presence of a residual splenic tissue after splenectomy [10–12]. Therefore, it may be a reasonable imaging option to distinguish splenic tissue from NETs or their metastases, contributing to the differential diagnosis of unclear ^68^Ga-DOTATATE PET/CT findings. The basic mechanism of this imaging technique is based on denaturated red blood cell uptake by the functioning spleen for the removal of abnormal erythrocytes.

The protocol for preparing red blood cells had been described as: drawing patient blood into a heparinized syringe, transferring it to a vacutainer tube followed by incubating five minutes with gentle mixing, adding EDTA solution and mixing again, centrifuging five minutes, withdrawing RBC after the cell fraction is separated from plasma, adding to saline-^99m^Tc pertechnetate solution and incubating for 15 min at 49 °C [13]. Scintigraphy and SPECT are required to be performed 30 min after the injection of prepared ^99m^Tc-labeled denaturated RBC with a high sensitivity and specificity when used together [14].

In a case series reported by Werner et al.*,*10 patients had been evaluated to whom ^68^Ga-DOTATOC PET/CT and ^99m^Tc-HDRBC scintigraphy-SPECT/CT were performed [10]. In 5 patients, surgery was discarded for the diagnosis because of the elevated tracer uptake within the lesions which were suggestive of the splenic tissue. One patient underwent surgery due to the absence of tracer uptake in ^99m^Tc-HDRBC scintigraphy-SPECT/CT and NET metastasis was shown histopatologically. In the remaining four patients, negative findings on ^99m^Tc-HDRBC scintigraphy-SPECT/CT has been considered as false negative and the most probable cause of DOTATOC accumulation has been still assumed to be a splenic tissue. The authors discussed about the two possible causes regarding the false negative imaging by ^99m^Tc-HDRBC scintigraphy-SPECT/CT which were the relatively small sizes of the lesions and respiratory-dependent excursions due to their close proximity to the diaphragm.

## Conclusions

As described in our presented case, ^99m^Tc-HDRBC scintigraphy-SPECT/CT is a useful nuclear medicine method to differentiate a NET in the pancreatic tail from accessory spleen at the splenic hilum which may avoid unnecessary surgeries in the presence of enhanced tracer uptake or vice versa.

## Data Availability

Not applicable.

## Abbreviations

DOTA: Tetraazacyclododecane-tetraacetic acid
DOTANOC: DOTA-1-Nal3-octreotide
DOTATATE: DOTA-d-Phe1-Tyr3-octreotate
DOTATOC: DOTA-d-Phe1-Tyr3-octreotide
EUS: Endoscopic ultrasonography
Ga: Gallium
NETs: Neuroendocrine tumors
PET/CT: Positron emission tomography/computed tomography
SPECT: Scintigraphy-single-photon emission computed tomography
SSTRs: Somatostatin receptors
^99m^Tc-HDRBC: ^99m^Tc-labelled heat-denaturated red blood cell
